# Supplementary figures and images for: Analysis of the saliva metabolic signature in patients with primary Sjögren’s syndrome
Source: PLoS One. 2022 Jun 2;17(6):e0269275. doi: 10.1371/journal.pone.0269275 (PMC9162338; doi:10.1371/journal.pone.0269275)

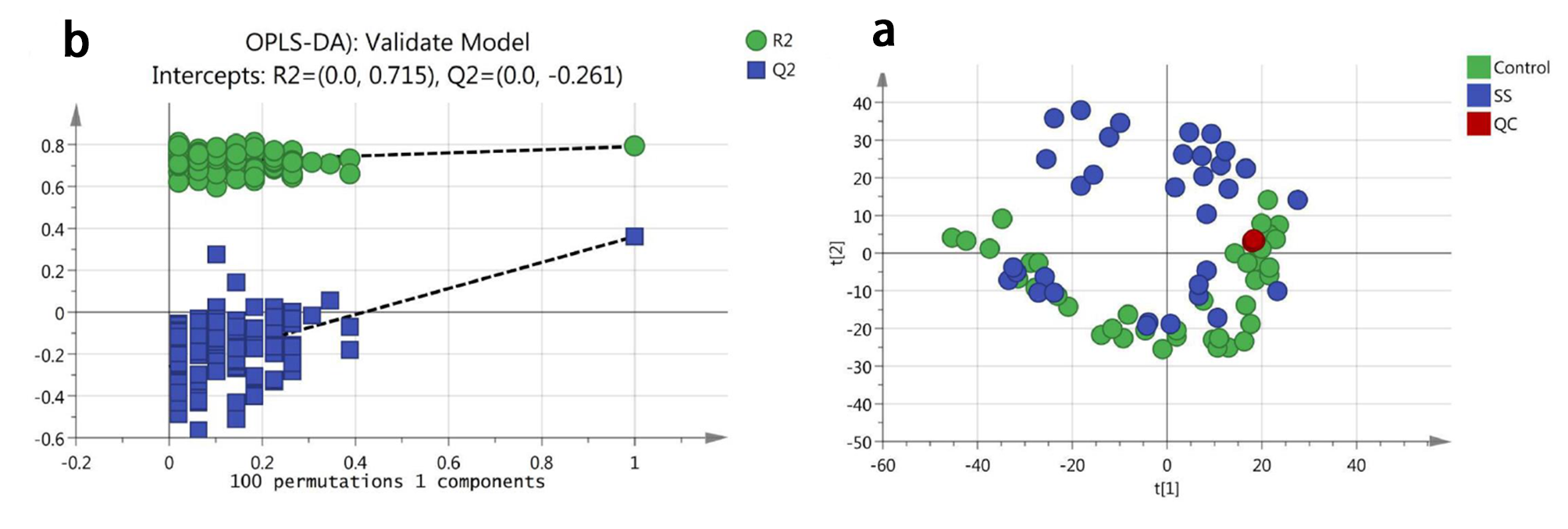

Supplement: S1 Fig — (a) Tight clustering of QC samples indicated good stability of the analysis. (b) Platform 100 times permutation tests for the OPLS-DA model. (TIF) [file pone.0269275.s001.tif]
