# Supplementary material for: Analysis of the saliva metabolic signature in patients with primary Sjögren’s syndrome
Source: PLoS One. 2022 Jun 2;17(6):e0269275. doi: 10.1371/journal.pone.0269275 (PMC9162338; doi:10.1371/journal.pone.0269275)
Supplement: S1 Table — Fold change > 1 indicates a relatively higher concentration of the marker present in pSS patients, whereas < 1 indicates a relatively lower concentration of the marker compared with healthy control subjects. (DOCX) [file pone.0269275.s002.docx]

**Supporting information**

**S1 Table. The metabolites related with pSS/control**

| **Name** | **Raw p.value** | **FDR** | **Fold change** | **VIP** | **AUC** |
| --- | --- | --- | --- | --- | --- |
| Aspartyl-Isoleucine | 4.50E-06 | 1.09E-04 | 9.94 | 2.26 | 0.88 |
| N-Phenylacetylaspartic acid | 5.96E-06 | 1.09E-04 | 8.44 | 2.01 | 0.87 |
| Phenylalanyl-Alanine | 8.99E-06 | 1.09E-04 | 8.11 | 1.95 | 0.87 |
| Isoleucyl-Lysine | 1.17E-05 | 1.09E-04 | 11.14 | 2.24 | 0.86 |
| Tyrosyl-Alanine | 1.53E-05 | 1.09E-04 | 18.24 | 2.42 | 0.86 |
| Tyrosyl-Glutamate | 1.97E-05 | 1.09E-04 | 12.62 | 2.04 | 0.85 |
| Leucyl-Glutamate | 2.24E-05 | 1.09E-04 | 34.16 | 2.61 | 0.85 |
| Isoleucyl-Arginine | 2.54E-05 | 1.09E-04 | 16.85 | 2.16 | 0.85 |
| Tryptophyl-Gamma-glutamate | 2.87E-05 | 1.09E-04 | 6.88 | 1.81 | 0.85 |
| Tyrosyl-Phenylalanine | 2.87E-05 | 1.09E-04 | 9.05 | 2.23 | 0.85 |
| Phenylalanyl-Gamma-glutamate | 4.66E-05 | 1.61E-04 | 11.25 | 2.16 | 0.84 |
| N6-Acetyl-L-lysine | 1.30E-04 | 2.36E-04 | 17.97 | 2.31 | 0.84 |
| Phenylalanyl-Aspartate | 5.90E-05 | 1.87E-04 | 32.31 | 2.41 | 0.84 |
| Asparaginyl-Valine | 8.33E-05 | 2.08E-04 | 19.32 | 2.15 | 0.83 |
| Isoleucyl-Valine | 8.33E-05 | 2.08E-04 | 16.59 | 2.28 | 0.83 |
| 8-Hydroxyadenine | 9.32E-05 | 2.08E-04 | 7.87 | 1.79 | 0.83 |
| Phenylalanyl-Asparagine | 9.32E-05 | 2.08E-04 | 6.79 | 1.71 | 0.83 |
| Phenylalanyl-Glutamate | 9.32E-05 | 2.08E-04 | 13.41 | 2.16 | 0.83 |
| N2-Succinyl-L-ornithine | 1.04E-04 | 2.20E-04 | 5.36 | 1.47 | 0.83 |
| Serotonin | 1.16E-04 | 2.33E-04 | 7.47 | 1.62 | 0.82 |
| 7-hydroxyoct-3-enoylglycine | 2.35E-04 | 3.44E-04 | 17.09 | 2.14 | 0.82 |
| Leucyl-Tyrosine | 1.30E-04 | 2.36E-04 | 12.13 | 2.18 | 0.82 |
| N-Acetylproline | 1.45E-04 | 2.50E-04 | 12.34 | 2.11 | 0.82 |
| Phenylalanyl-Valine | 1.61E-04 | 2.67E-04 | 17.41 | 2.24 | 0.82 |
| Oxypurinol | 1.80E-04 | 2.73E-04 | 9.01 | 1.93 | 0.82 |
| Threoninyl-Valine | 1.80E-04 | 2.73E-04 | 13.87 | 2.11 | 0.82 |
| Isoleucyl-Leucine | 3.82E-04 | 4.84E-04 | 7.59 | 1.56 | 0.81 |
| Threoninyl-Isoleucine | 2.73E-04 | 3.70E-04 | 14.48 | 2.02 | 0.81 |
| Valyl-Valine | 2.73E-04 | 3.70E-04 | 13.37 | 2.20 | 0.81 |
| Asparaginyl-Leucine | 3.02E-04 | 3.96E-04 | 10.74 | 1.80 | 0.81 |
| gamma-Glutamylleucine | 5.47E-04 | 6.70E-04 | 12.60 | 1.75 | 0.79 |
| Hydroxyprolyl-Lysine | 6.02E-04 | 6.93E-04 | 10.17 | 1.55 | 0.79 |
| Tryptophyl-Isoleucine | 6.02E-04 | 6.93E-04 | 5.93 | 1.56 | 0.79 |
| Glutaminylvaline | 7.98E-04 | 8.92E-04 | 5.01 | 1.41 | 0.79 |
| Alpha-Linolenoyl ethanolamide | 1.15E-03 | 1.25E-03 | 3.15 | 1.70 | 0.78 |
| Tyrosyl-Asparagine | 1.37E-03 | 1.45E-03 | 3.25 | 1.30 | 0.77 |
| Tyrosyl-Hydroxyproline | 1.50E-03 | 1.54E-03 | 3.24 | 1.43 | 0.77 |
| Tyrosyl-Valine | 2.06E-02 | 2.06E-02 | 3.78 | 1.00 | 0.70 |

Fold change > 1 indicates a relatively higher concentration of the marker present in pSS patients, whereas < 1 indicates a relatively lower concentration of the marker compared with healthy control subjects.
