# Supplementary material for: Analysis of the saliva metabolic signature in patients with primary Sjögren’s syndrome
Source: PLoS One. 2022 Jun 2;17(6):e0269275. doi: 10.1371/journal.pone.0269275 (PMC9162338; doi:10.1371/journal.pone.0269275)
Supplement: S2 Table — Note: The sensitives and specificities were calculated at their best cutoff points. (DOCX) [file pone.0269275.s003.docx]

**S2 Table. Receiver operating characteristic (ROC) analysis of potential pSS biomarkers**

|  | **Name** | **AUC** | **Sensitivity** | **Specificity** |
| --- | --- | --- | --- | --- |
|  | Phenylalanyl-Alanine | 0.87 | 0.8 | 0.84 |
| Discovery set | Asparaginyl-Valine | 0.83 | 0.80 | 0.88 |
|  | Oxypurinol | 0.82 | 0.80 | 0.88 |
|  | Phenylalanyl-Alanine | 0.75 | 0.75 | 0.85 |
| Validation set | Asparaginyl-Valine | 0.74 | 0.58 | 0.85 |
|  | Oxypurinol | 0.73 | 0.75 | 0.70 |

Note: The sensitivities and specificities were calculated at their best cutoff points
